# Coordination analysis of flood-sediment transportation, eco-environment, and socio-economy coupling in the governance of the Yellow River Basin system

**DOI:** 10.1038/s41598-024-58759-4

**Published:** 2024-04-06

**Authors:** Gaolei Zhao, Shimin Tian, Enhui Jiang, Yongcai Jing, Rongxu Chen, Xin Wang, Yang Zhang

**Affiliations:** Henan Key Laboratory of YB Ecological Protection and Restoration, Yellow River Institute of Hydraulic Research, YRCC, Zhengzhou, 450003 China

**Keywords:** Coupling coordination, Spatio-temporal heterogeneity, Flood-sediment transportation, Eco-environment, Socio-economy, Yellow River Basin, Environmental impact, Environmental impact, Sustainability, Hydrology

## Abstract

The watershed system has a complex game relationship between the benign operation and coordinated development of various elements of flood-sediment transportation, eco-environment, and socio-economy (FES). With the increasing breadth, depth, and intensity of human activities in watersheds, it is urgent to coordinate the FES. The relationship of water–sediment in the Yellow River Basin (YRB) is complex, with a prominent contradiction in water supply and a fragile ecosystem. This research tries to build a comprehensive evaluation model for FES and explore the complex interaction between FES in the YRB from 2000 to 2020. The results demonstrated that (1) the comprehensive flood-sediment transportation index (CFTI) and comprehensive eco-environment index (CEI) presented fluctuating growth. In contrast, the comprehensive socio-economy index (CSI) revealed a linear growth trend. The CFTI of Sanmenxia, CEI of Toudaokuan, and CSI of Ningxia had the highest growth rates, with 36.03%, 6.48%, and 107.5%, respectively. (2) FES's positive and negative effects were alternating, with heterogeneity in both time and space. (3) The coupling coordination degree (CCD) in the YRB indicated an increasing trend, ranging from 0.53 to 0.87, from reluctantly coordinated development to good coordinated development. The lagging subsystem was CFTI (2000–2001 and 2008–2020) and CSI (2002–2007), and the CEI was not lagging. (4) Exploratory Spatial Data Analysis (ESDA) demonstrated significant differences in the CCD of the YRB, and areas with similar CCD within the basin tend to be centrally distributed in space. At the same time, there was negative spatial autocorrelation in coordination. The results provide a scientific theoretical and methodological framework for strategic research on the YRB system's governance, protection, and management.

## Introduction

The World Cities Report 2022: Envisioning the Future of Cities, released by the United Nations, demonstrated that the global urban population increased from 29.5% in 1950 to 56.2% in 2021, and the pace of world urbanization will continue to accelerate in the next 30 years, and the world urbanization rate will increase from 56.2% in 2021 to 68% in 2050^1^. In China, the urbanization rate of the permanent population exceeded 65.2% in 2022^2^ and will reach 65.5% in 2025^3^ and 70% in 2050^4^. Northam^5^ pointed out that countries or regions with 30–70% urbanization rates are in an accelerated stage of development. At this stage, the required water resources^6^ significantly reduced environmental carrying capacity, leading to a series of problems^7–10^, such as water quality degradation^11^, ecological and environmental issues^12–16^, water scarcity, and pollution^17,18^ posed a significant threat to sustainable development^19^. The rapid economic and social development coupled with the reduction of water resources has attracted great attention from the public and scientific researchers on environmental and ecological issues^20–22^.

Since the beginning of the twenty-first century, the intensity of human activities' demand for natural ecosystems has been continuously increasing, leading to serious imbalances in natural ecosystems, resulting in prominent environmental problems such as global warming, food crisis, water resource shortage, ecosystem degradation, decreased biodiversity, land desertification, and marine pollution^23,24^. The YRB is the core birthplace of ancient Chinese civilization, supporting over 150 million people and 15% of China's farmland^25^. In recent decades, the sedimentation, water quality deterioration, and river flow reduction in the YRB have significantly increased^26,27^. Against the backdrop of global climate change, high-intensity urbanization, and industrial development, a series of issues affecting sustainable urbanization construction have emerged in the YRB, including water resource shortage, water environment pollution, land desertification, and fragile ecological environment, posing a serious threat to the ecological environment quality of the basin^28^. The YRB is the river with the highest sediment content in the world, the second largest river in China, and the mother river of the Chinese nation, which has always been weak and sickly, with frequent floods, threats from floods, a fragile ecological environment in the basin, difficult water resource protection situations, and the need to improve development quality^29^. The ecological protection of the YRB is of great significance for the development and stability of China^25^. In recent years, the ecological protection of the YRB has received great attention from government departments^30^.

A watershed is a natural geographic system formed based on water systems, connecting natural elements such as mountains, rivers, forests, fields, lakes, and grasses as a unified whole^31,32^. The watershed provides important natural resources and a development environment for socio-economic development. Still, excessive and substandard pollutants are discharged into the watershed during the socio-economic development process, causing serious pollution that destroys the stability of the watershed ecosystem^33^. Water is a fundamental natural and strategic resource, and ecology is an important link to maintaining the relationship between humans and nature^34^. The YRB, the region with the strongest interaction between humans and nature, strongly depends on the healthy life of rivers for socio-economic development. Based on the functional attributes of the YRB, the YRB system can be defined as a composite system with the ultimate goals of maintaining the basic functions of rivers (flood and sediment transport), sustainable socio-economic development, and effective protection of the ecological environment^35^. Therefore, it is necessary to quantify the relationship between FES in the YRB to support scientific decision-making for the governance of the YRB system.

The coupling theory originated in the field of physics^36^ and is a phenomenon where two or more systems interact and influence each other^6,37,38^, reflecting the strength of the interaction between the two parties and regulating and controlling various coupling processes^39^. Since its introduction, coupling theory has expanded to many fields, including environmental science, management science, ecology, and sociology^40^, playing a crucial role in studying the relationships between two or more systems^41^. The coupling degree (CD) is used to analyze the complex coupling relationships of multiple systems quantitatively. CD refers to measuring the degree of mutual correlation and internal effects between different systems, including control relationships, activation relationships, transmission relationships, etc^42^. The CD describes the degree of collaboration between sequence parameters during the development of a system. The key to the orderly development of a system is the synergistic effect between various subsystems within the system^43^. CD can reflect the mutual influence and connection between multiple subsystems, and the higher (lower) the CD, the higher (lower) the degree of correlation between subsystems, and the more stable (disordered) the system state^44^.

Since the CD can only represent the degree of mutual influence and interaction between different systems, there may be a phenomenon of false coupling (both systems have low comprehensive levels but high CD) at a specific moment, so a coupling coordination degree model (CCDM) needs to be introduced^45^. The concept of CCD co-scheduling^46^ was firstly proposed by Chinese scholar Yang Shihong in the 1990s, based on previous theories, and was inherited and developed by later scholars. CCD is a phenomenon originating from system theory, emphasizing the close connections and complex interactions between different subsystems, which can explain the sustainable development of the system^47,48^. CCD is an indicator that measures the degree of coupling between various subsystems within a system and can also demonstrate the level of coordinated development between each subsystem. The CCD index has a complete evaluation plan and evaluation criteria^49^, and the CCDM was applied to elucidate the interrelationships between subsystems and the coordination level of the system composed of several subsystems^50^. This model is easy to calculate and has intuitive results, so it is widely used to study the CCD development level between multiple systems such as economy, agriculture, urbanization, and ecology^51–55^. The main research methods for quantitative modeling of multiple system coupling relationships include the STIRPAT model^56,57^, coupling coordination degree model^45^, multi-agent model^58^, and big data and urban computing models^59–62^.

At present, there are mainly systematic studies on the YRB, including water-energy-food^63,64^, low-carbon development-eco-environment^65^, economic growth-urbanization construction-water resources-industrial development^66^, water utilization-industrial development-ecological welfare^67^, production-living-ecological spaces^68^. The methods used in these studies are all CCDM. The CCDM is widely used to explore the coupling relationship and degree of interaction between different systems, but there is no research on the CCD relationship between FES. The three factors of FES are key driving forces for promoting ecological protection and high-quality development in the YRB, all of which are essential. From the systems theory perspective, these three factors together form a holistic system, each of which complements other factors.

The YRB covers different natural and geographical conditions, socio-economic development models, ecological environment types, and water demand. On the other hand, the evolution characteristics of the YRB channel also have significant spatial differences. The comprehensive management of the YRB needs to consider various factors such as nature, society, ecology, sediment, and river channels^69^. Therefore, studying the coupling relationship between FES in the YRB is crucial and has an important theoretical and practical significance for watershed governance and environmental pollution control in China's urbanization process. This paper attempts to treat the FES of the YRB as subsystems of the entire basin. By constructing a CCDM of FES systems in the basin, the CCD relationship between FES systems in the YRB is explored. At the same time, the ESDA^36^ was applied to evaluate the CCD of FES in the YRB. This study aims to (1) calculate the comprehensive FES index to explore the FES development status of the YRB. (2) Analyze the coupling and coordination between FES in various regions by CCDM. (3) Test the spatio-temporal heterogeneity to reveal the bidirectional relationships between FES by Geographically and Temporally Weighted Regression (GTWR) in the YRB from 2000 to 2020. (4) Analysis of the disparities in the CCD of the FES based on ESDA.

## Materials and methods

### Study area

**Figure 1:**
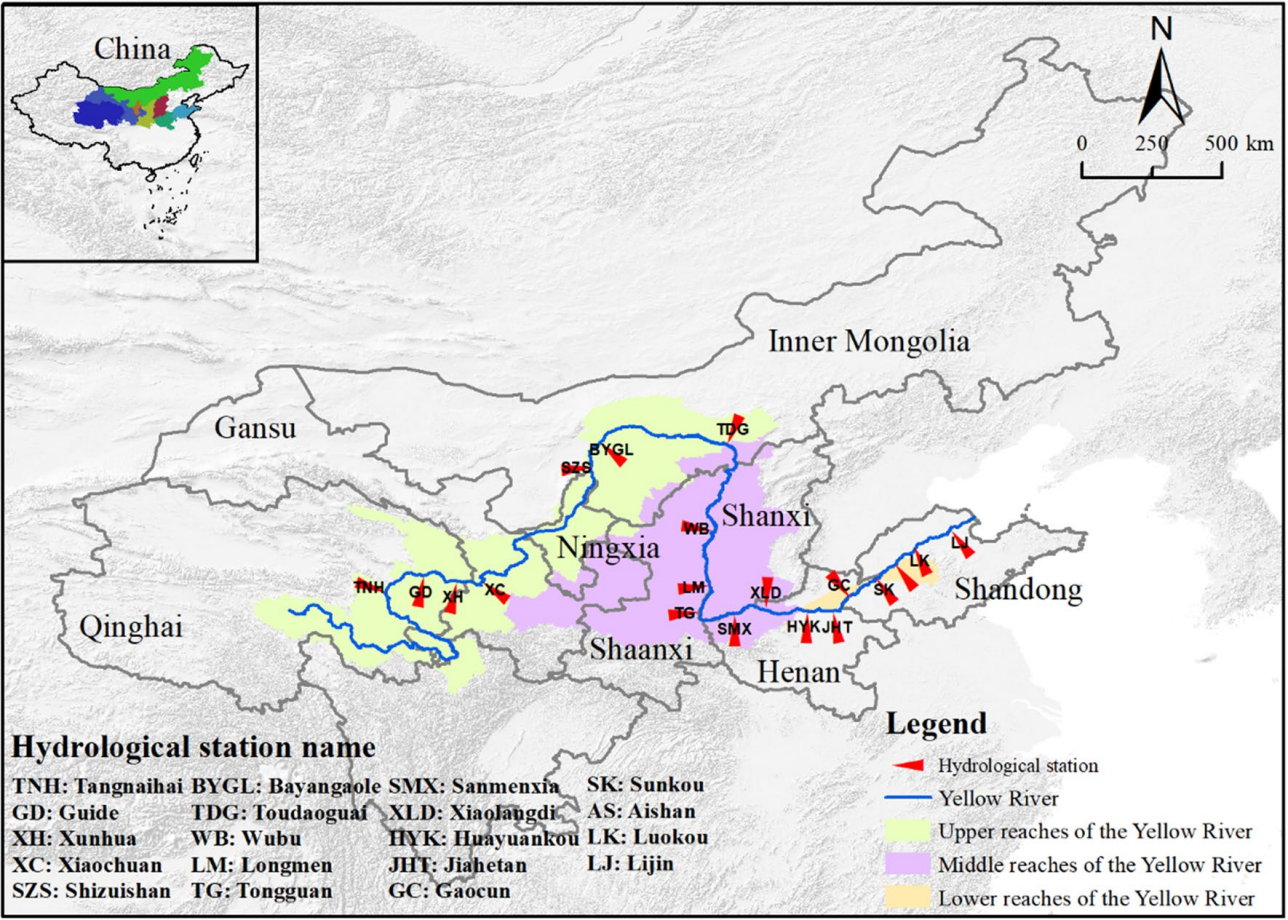
The location of the study area. The map was generated by the authors with the help of ArcGIS 10.2 (https://support.esri.com/en/download/2093) and does not require any permission from anywhere.

The Yellow River originates from the Qinghai-Tibet Plateau in Qinghai. Its mainstream flows through Qinghai, Sichuan, Gansu, Ningxia, Inner Mongolia, Shanxi, Shaanxi, Henan, and Shandong provinces and flows into the Bohai Sea in Dongying. The total length of the YR is 5464 km, with a drop of 4480 m and a drainage area of 795,000 km^2^. The YRB is located between 95°53′–119°5′ E and 32°10′–41°50′ N (Fig. 1). The map in Fig. 1 was prepared by the co-authors with the help of ArcGIS 10.2 (https://support.esri.com/en/download/2093). Above Hekou is the upper reaches of the YRB, with a length of 3471.6 km and a drainage area of 428,000 km^2^. From Hekou to Taohuayu is the middle reaches of the YRB, with a length of 1206.4 km and a drainage area of 344,000 km^2^. It is downstream from Taohuayu to the estuary, with a length of 785.6 km and a catchable area of 23,000 km^2^.

The YRB is in the northern part of the East Asian Sea land monsoon region. The average annual precipitation in the YRB over the years is 476 mm, and the seasonal distribution of precipitation is very uneven, mainly concentrated in summer and autumn (from June to September). The distribution of precipitation shows a decreasing trend from southeast to northwest, with more in the east and less in the west, and more in the south and less in the north. The annual average temperature in the YRB ranges from – 4 to 14℃, with a general trend of high in the south and low in the north, high in the east and low in the west. In 2020, the watershed's population was 320 million, the urbanization rate was 53.42%, the total water resources were 649.08 billion cubic meters, and the gross domestic product was 20.83 trillion yuan. The proportions of the primary, secondary, and tertiary industries are 7.42%, 41.14%, and 51.44%, respectively.

### Yellow River Basin system

**Figure 2:**
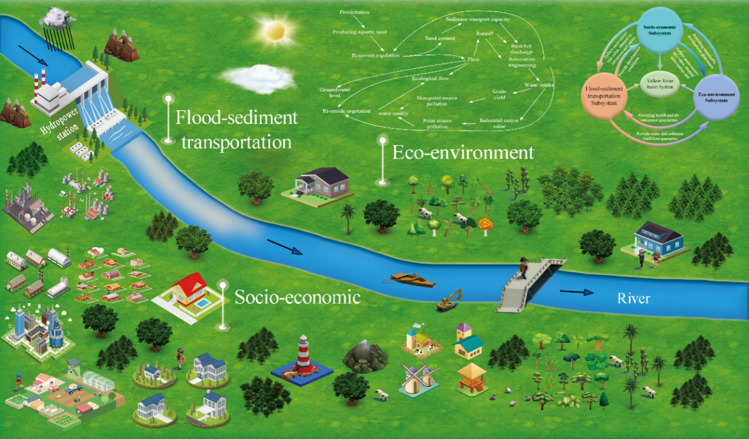
Schematic diagram of the interaction between flood dimension transportation, eco-environment, and socio-economy of the Yellow River Basin.

Schum^70^ proposed that according to the natural state of a river, the entire river can be divided into three subsystems from upstream to downstream: the catchment basin subsystem, the river channel subsystem, and the estuarine delta subsystem. Jiang et al.^35^ divided the watershed system into three major subsystems based on river functions: flood-sediment transportation subsystem, eco-environment subsystem, and socio-economic subsystem. This study adopted the watershed subsystem classification method proposed by Jiang et al.^35^, and Fig. 2 shows a schematic diagram of the interaction between the three subsystems in the YRB. The flood-sediment transportation system includes the natural structure of rivers, such as riparian zones, riverbeds, water bodies, and wading works, which is related to the transport of river water and sand materials and directly impacts the socio-economy and eco-environment. The socio-economic subsystem includes indicators such as GDP, grain yield, and other indicators that reflect the supporting function of rivers for socio-economic development, such as water diversion. The composition of the socio-economic subsystem here is limited to content related to the interaction between rivers and socio-economic factors. It does not include content that is completely part of the social system, such as history, culture, and social relations. The eco-environment subsystem consists of the biological communities, habitats, vegetation, and other components related to the ecological environment in rivers and riverfront areas. Under the theoretical framework of the YRB system, each subsystem has its structure, function, and composition and can be characterized by a series of characteristic indicators during the research process.

### Data resources and preprocessing

The raw data of different indicators have significant differences in amplitude and scale, and the data lacks comparability. Therefore, it is necessary to standardize the raw data for extreme differences^71^.

The socio-economic statistical data are from the past Statistical Yearbook and Water Resources Bulletin of Qinghai, Gansu, Ningxia, Inner Mongolia, Shanxi, Shaanxi, Henan, and Shandong from 2000 to 2020. The flood-sediment transportation and eco-environment statistical data are initially from the YRB Hydrological Yearbook from 2000 to 2020.

1 $$x^{\prime}_{ij} = \left\{ \begin{aligned} & \frac{{x_{ij} - \mathop {\min }\limits_{1 \le i \le m} \left( {x_{ij} } \right)}}{{\mathop {\max }\limits_{1 \le i \le m} \left( {x_{ij} } \right) - \mathop {\min }\limits_{1 \le i \le m} \left( {x_{ij} } \right)}}{\text{ Positive indicator}} \hfill \\ & \frac{{\mathop {\max }\limits_{1 \le i \le m} \left( {x_{ij} } \right) - x_{ij} }}{{\mathop {\max }\limits_{1 \le i \le m} \left( {x_{ij} } \right) - \mathop {\min }\limits_{1 \le i \le m} \left( {x_{ij} } \right)}}{\text{ Negative indicator}} \hfill \\ \end{aligned} \right.$$

where *x*_*ij*_ and $x^{\prime}_{ij}$ are the primitive and standardized values of the indicator *j* in the year *i*, respectively. *max*(*x*_*ij*_) and *min*(*x*_*ij*_) are the maximum and minimum values of the indicator *j* in the year *i*, respectively.

### Analysis methods

#### Construction of the indicator system

The flood-sediment transportation subsystem is related to the basic functions of river material transport and flood and sand transport. The eco-environment subsystem is associated with the quality and part of the ecological environment within the river system. In contrast, the socio-economic subsystem is related to the supporting role of the river in the socio-economic development of the river area and the degree of dependence of the latter on the former^35^.

The flood-sediment transportation subsystem includes river hydrological processes, the carrying capacity of rivers, and the regulation capacity of reservoirs. The constraint factors can be divided into runoff and sediment, including indicators reflecting runoff characteristics, flow characteristics, and sediment characteristics. The constraint factors in the eco-environment subsystem are based on the ecological water demand of typical river sections in the YRB. In the socio-economic subsystem, the water supply in the YRB is mainly irrigation, so in selecting socio-economic constraints, factors that characterize agricultural production are emphasized, including grain production, primary output value, sowing area of grain crops, GDP, and water intake. Table 1 is the index system.

**Table 1:** The indicator system used to evaluate FES in the YRB.

Subsystem	Index	Unit	Direction
Flood-sediment transportation	F1 Annual average flow	m^3^/s	+
	F2 Average flow during flood season	m^3^/s	+
	F3 Annual maximum flow	m^3^/s	+
	F4 Annual minimum flow	m^3^/s	+
	F5 3-day maximum average flow	m^3^/s	+
	F6 7-day maximum average flow	m^3^/s	+
	F7 Annual average sediment concentration	kg/m^3^	+
	F8 Average sediment concentration during flood season	kg/m^3^	+
	F9 Annual sediment discharge	10^8^t	+
	F10 Sediment transport during flood season	10^8^t	+
Eco-environment	E1 Suitable flow guarantee rate from January to March	%	+
	E2 Low limit traffic guarantee rate from January to March	%	+
	E3 Suitable flow guarantee rate from May to June	%	+
	E4 Suitable number of flow pulses from April to June	–	+
	E5 Number of low limit flow pulses from April to June	–	+
	E6 Suitable flow guarantee rate from November to December	%	+
	E7 Low limit traffic guarantee rate from November to December	%	+
Socio-economic	S1 Grain production	10^4^ tons	+
	S2 Primary output value	In yuan	+
	S3 Sowing area of grain crops	10^4^ ha	+
	S4 GDP	In yuan	+
	S5 Water intake	10^8^ m^3^	−

Water intake refers to the total amount of water taken by various types of water users outside the river, including domestic water, industrial water, agricultural water, and artificial ecological environment replenishment.

#### Calculation of the comprehensive index

The entropy method is often used to determine the weight of the CCDM^72^.

2 $$s_{ij} = {{x_{ij} }/ {\sum\limits_{i = 1}^{n} {x_{ij} } }}$$

3 $$e_{j} = - \frac{1}{\ln \left( n \right)}\sum\limits_{i = 1}^{n} {s_{ij} \ln s_{ij} } \, \left( {0 \le e_{j} \le 1} \right)$$

4 $$g_{j} = 1 - e_{j}$$

5 $$\omega_{j} = {{g_{j} } /{\sum\limits_{j = 1}^{m} {g_{j} } }}$$

6 $$u_{ij} = \sum\limits_{i = 1,j = 1}^{n,m} {\omega_{j} } x_{ij}^{\prime }$$

where *S*_*ij*_ is the proportion of the indicator *j* in the year *i*; *e*_*j*_ is the entropy of each indicator; *g*_*j*_ is the entropy redundancy; $\omega_{j}$ is the weight of each indicator *j*; *u*_*ij*_ is the comprehensive effectiveness of these subsystems, including CFTI, CEI, CSI; *n* is years, and *m* is the number of indicators.

#### Coupling coordination degree model

7 $$CD = \left\{ {{{\left( {u_{1} \cdot u_{2} \cdot u_{3} } \right)}/{\left[ {{{\left( {u_{1} + u_{2} + u_{3} } \right)} / 3}} \right]}}^{3} } \right\}^{\frac{1}{3}}$$

8 $$CCD = \sqrt {CD \times T}$$

9 $$T = \alpha u_{1} \times \beta u_{2} \times \gamma u_{3}$$

where $u_{1}$, $u_{2}$, and $u_{3}$ respectively represent CFTI, CEI, and CSI. *T* is the comprehensive coordination index of systematic FES. $\alpha$, $\beta$ and $\gamma$ are the undetermined coefficient. Since the urban subsystem and water environment subsystem are equally important, this paper took $\alpha = \beta = \gamma = \frac{1}{3}$^67^. Referring to the research results of Wang et al.^73^, this study divided the CCD results into four levels (Table 2).

**Table 2:** The classification of CCD.

Category	Coordination	Subcategory	Systematic exponential comparison
Good coordinated development (GCD)	0.8 ≤ CCD ≤ 1	GCD type with lagging flood-sediment transportation GCD type with lagging eco-environment GCD with lagging socio-economy GCD type of flood-sediment transportation, eco-environment and socio-economy	CEI-CFTI ≥ 0.1 or CSI-CFTI ≥ 0.1 CFTI-CEI ≥ 0.1 or CSI-CEI ≥ 0.1 CFTI-CSI ≥ 0.1 or CEI- CSI ≥ 0.1 Difference between any two items CFTI, CEI, and CSI is < 0.1
Moderate coordinated development (MCD)	0.7 ≤ D < 0.8	MCD type with lagging flood-sediment transportation MCD type with lagging eco-environment MCD type with lagging socio-economy MCD type of flood-sediment transportation, eco-environment and socio-economy	CEI-CFTI ≥ 0.1 or CSI-CFTI ≥ 0.1 CFTI-CEI ≥ 0.1 or CSI-CEI ≥ 0.1 CFTI-CSI ≥ 0.1 or CEI- CSI ≥ 0.1 Difference between any two items CFTI, CEI, and CSI is < 0.1
Primary coordinated development (PCD)	0.6 ≤ D < 0.7	PCD type with lagging flood-sediment transportation PCD type with lagging eco-environment PCD type with lagging socio-economy PCD type of flood-sediment transportation, eco-environment and socio-economy	CEI-CFTI ≥ 0.1 or CSI-CFTI ≥ 0.1 CFTI-CEI ≥ 0.1 or CSI-CEI ≥ 0.1 CFTI-CSI ≥ 0.1 or CEI- CSI ≥ 0.1 Difference between any two items CFTI, CEI, and CSI is < 0.1
Reluctantly coordinated development (RCD)	0.5 ≤ D < 0.6	RCD type with lagging flood-sediment transportation RCD with lagging eco-environment RCD with lagging socio-economy RCD type of flood-sediment transportation, eco-environment and socio-economy	CEI-CFTI ≥ 0.1 or CSI-CFTI ≥ 0.1 CFTI-CEI ≥ 0.1 or CSI-CEI ≥ 0.1 CFTI-CSI ≥ 0.1 or CEI- CSI ≥ 0.1 Difference between any two items CFTI, CEI, and CSI is < 0.1
Near dysfunction decline (NDD)	0.4 ≤ D < 0.5	NDD type with lagging flood-sediment transportation NDD type with lagging eco-environment NDD type with lagging socio-economy NDD type of flood-sediment transportation, eco-environment and socio-economy	CEI-CFTI ≥ 0.1 or CSI-CFTI ≥ 0.1 CFTI-CEI ≥ 0.1 or CSI-CEI ≥ 0.1 CFTI-CSI ≥ 0.1 or CEI- CSI ≥ 0.1 The difference between any two items CFTI, CEI, and CSI is < 0.1
Lightly dysfunctional decline (LDD)	0.3 ≤ D < 0.4	LDD type with lagging flood-sediment transportation LDD type with lagging eco-environment LDD type with lagging socio-economy LDD type of flood-sediment transportation, eco-environment and socio-economy	CEI-CFTI ≥ 0.1 or CSI-CFTI ≥ 0.1 CFTI-CEI ≥ 0.1 or CSI-CEI ≥ 0.1 CFTI-CSI ≥ 0.1 or CEI- CSI ≥ 0.1 Difference between any two items CFTI, CEI, and CSI is < 0.1
Moderate dysfunctional decline (MDD)	0.2 ≤ D < 0.3	MDD type with lagging flood-sediment transportation MDD e type with lagging eco-environment MDD e type with lagging socio-economy MDD type of flood-sediment transportation, eco-environment and socio-economy	CEI-CFTI ≥ 0.1 or CSI-CFTI ≥ 0.1 CFTI-CEI ≥ 0.1 or CSI-CEI ≥ 0.1 CFTI-CSI ≥ 0.1 or CEI- CSI ≥ 0.1 Difference between any two items CFTI, CEI, and CSI is < 0.1
Severe dysregulation decline (SDD)	0 < D < 0.2	SDD type with lagging flood-sediment transportation SDD type with lagging eco-environment SDD type with lagging socio-economy SDD type of flood-sediment transportation, eco-environment and socio-economy	CEI-CFTI ≥ 0.1 or CSI-CFTI ≥ 0.1 CFTI-CEI ≥ 0.1 or CSI-CEI ≥ 0.1 CFTI-CSI ≥ 0.1 or CEI- CSI ≥ 0.1 Difference between any two items CFTI, CEI, and CSI is < 0.1

#### Geographically and temporally weighted regression

GTWR can more intuitively display the geostatistical relationships of variables within each sample area at any time node, effectively reflecting the evolutionary relationships of variables in spatiotemporal scenarios^74^.

10 $$y_{i} = \beta_{0} \left( {u_{i} ,\nu_{i} ,t_{i} } \right) + \sum\limits_{k = 1}^{p} {\beta_{k} \left( {u_{i} ,\nu_{i} ,t_{i} } \right)} x_{ik} + \varepsilon_{i} \;\;i = 1,2, \ldots ,n$$

where $\left( {u_{i} ,v_{i} ,t_{i} } \right)$ represents the spatiotemporal three-dimensional coordinates of the *i*-sample point, *n* represents the number of sample points and $\beta_{k} \left( {u_{i} ,v_{i} ,t_{i} } \right)$ represents the regression coefficient of the *k* independent variable of the *i*-sample point. $\varepsilon_{i}$ represents the random error of the *i*-sample point.

#### Exploratory spatial data analysis

ESDA can be applied to global and local spatial agglomeration and anomalies, revealing the spatial interaction mechanism between different systems^36^. Global Moran’s I is the spatial correlation pattern of specific attribute values in the study area^75^ between − 1 and 1.

11 $$Moran^{\prime}s \, I{ = }\frac{n}{{\sum\nolimits_{i}^{n} {\sum\nolimits_{j}^{n} {w_{ij} } } }} \times \frac{{\sum\nolimits_{i}^{{}} {\sum\nolimits_{j}^{{}} {w_{ij} \left( {x_{i} - \overline{x} } \right)\left( {x_{j} - \overline{x} } \right)} } }}{{\sum\nolimits_{i}^{{}} {\left( {x_{i} - \overline{x} } \right)^{2} } }}$$

where *w*_*ij*_ represents the element of the two-dimensional spatial weight matrix, *n* represents the Number of regional units. *x*_*i*_ and *x*_*j*_ represent the observed values of *i*_*th*_ and *j*_*th*_ prefecture-level systems, respectively, and $\overline{x}$ represent the average value of the experimental values of prefecture-level systems.

## Results and analysis

### Changes in CFTI, CEI, and CSI of the Yellow River Basin

**Figure 3:**
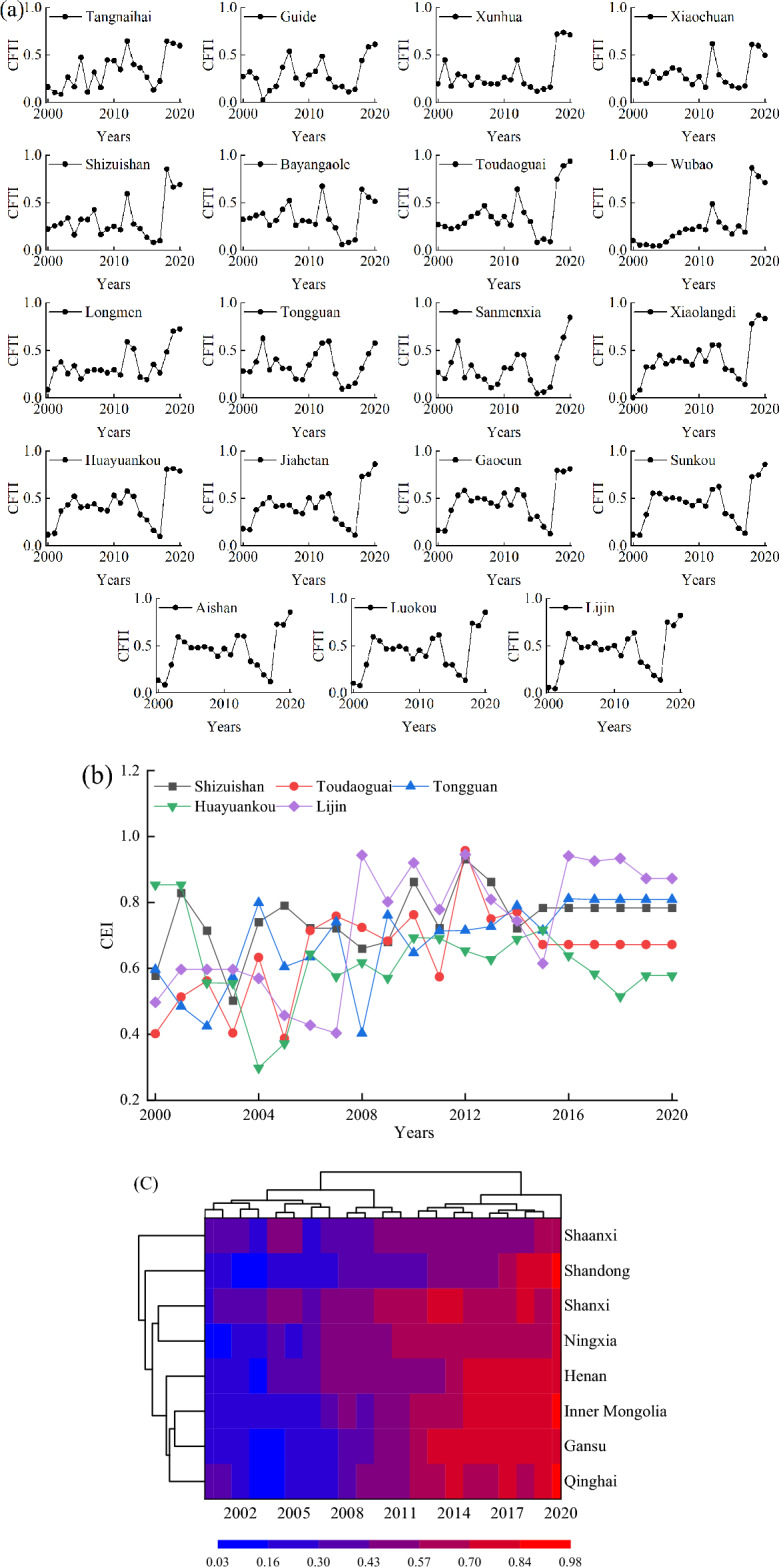
Changes in CFTI (**a**) and CEI (**b**), heatmap of changes in CSI (**c**) of the Yellow River Basin from 2000 to 2020.

Figure 3a shows the apparent change trend of CFTI in the YRB from 2000 to 2020. During the study period, the CFTI of each hydrological station varied frequently and had significant differences. The overall trend of CFTI from 2000 to 2014 was increasing, with a decrease from 2015 to 2017 and an increase from 2018 to 2020. Sanmenxia's CFTI presented the most significant change, increasing from 0.01 to 0.84, with an increased rate of 107.5%, followed by Lijin, Shizhuishan, and Huayuankou, with growth rates of 52.3%, 43.96%, and 41.46%, respectively.

The CEI of each hydrological station changes frequently and shows an overall increasing trend (Fig. 3b). Toudaoguai's CEI revealed the most significant change, increasing from 0.4 to 0.67, with an increased rate of 6.48%, followed by Lijin, Tongguan, Shizuishan, and Huayuankou, with growth rates of 6.46%, 4.66%, 3.22%, and 0.61%, respectively.

As can be seen from the color change in Fig. 3c, CSI gradually increased in the YRB from 2000 to 2020. Among them, Ningxia had the most significant change (36.03%), followed by Shandong, Inner Mongolia, Gansu, and Henan, with growth rates of 23.27%, 24.51%, 22.5%, and 24.69%, respectively.

### The spatial–temporal heterogeneity between CFTI, CEI and CSI

Because there are three subsystems in the FES system, GTWR analysis is divided into two cases: (1) One explanatory variable and one dependent variable; (2) Two Explanatory variables and one dependent variable.

#### One explanatory variable and one dependent variable

**Figure 4:**
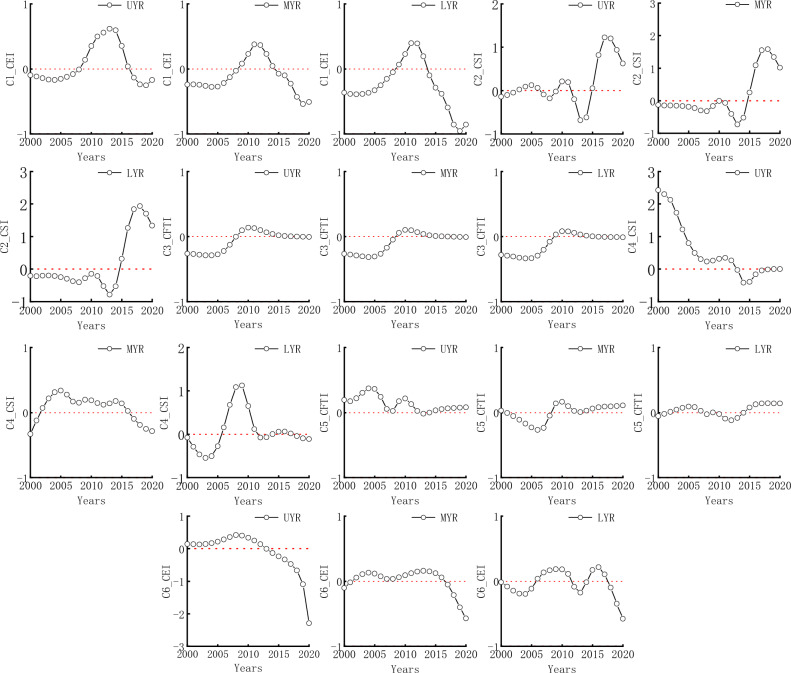
The impact of one explanatory variable on one dependent variable from 2000 to 2020.

For one explanatory variable and one dependent variable, Table 3 shows the other parameters. The feedback of different systems in the YRB from 2000 to 2020 is shown in Fig. 4. This model had good explanatory power and can effectively describe the relationship between one explanatory variable and one dependent variable. There was spatiotemporal heterogeneity between the three subsystems in the upper, middle, and lower reaches of the YRB and in terms of time.

**Table 3:** GTWR model results of capturing two of the three subsystems as expansive and dependent variables, respectively.

Dependent variable Explanatory variable	CFTI CEI (C1)	CFTI CSI (C2)	CEI CFTI (C3)	CEI CSI (C4)	CSI CFTI (C5)	CSI CEI (C6)
Bandwidth	0.115	0.115	0.196	0.115	0.115	0.115
Residual squares	0.790	0.839	0.419	0.298	0.093	0.092
Sigma	0.112	0.115	0.082	0.069	0.038	0.038
AICc	− 51.746	− 56.025	− 116.872	− 104.412	− 171.533	− 167.434
R^2^	0.643	0.621	0.548	0.678	0.960	0.960
Spatio-temporal distance ratio	4.849	4.827	4.486	1.658	1.157	1.399
Trace_of_SMatrix	15.132	12.852	7.167	17.357	18.897	19.894

The impact of CEI on CFTI and CFTI on CEI is consistent in the upper reaches of the Yellow River (UYR), middle reaches of the Yellow River (MYR), and lower reaches of the Yellow River (LYR). The CEI has a positive impact on CFTI from 2000–2008 and 2017–2020 and a negative impact from 2009–2016; The effect of CFTI on CEI was positive from 2009–2017 and negative from 2000–2008 and 2018–2020.

There were differences in the impact of CSI on CFTI, CSI on CEI, CFTI on CSI, and CEI on CSI in the UYR, MYR and LYR. The effects of CSI on CFTI in the UYR are negative (2000–2002), positive (2003–2006), negative (2007–2009), positive (2010–2011), negative (2012–2014), positive (2015–2020); in the MYR, negative (2000–2014), positive (2015–2020); in the LYR, negative (2000–2014), positive (2015–2020).

The impact of CSI on CEI in the UYR was positive (2000–2014), negative (2015–2020); in the MYR, negative (2000–2001), positive (2002–2016), negative (2016–2020); in the LYR, negative (2000–2005), positive (2006–2011), negative (2012–2013), positive (2014–2017), negative (2018–2020).

The impact of CFTI on CSI in the UYR was positive (2000–2012), negative (2013–2014), positive (2015–2020); in the MLR, negative (2000–2008), positive (2009–2020); in the LYR, positive (2000–2007), negative (2008), positive (2009), negative (2010–2014), positive (2015–2020).

The impact of CEI on CSI in the UYR was positive (2000–2013), negative (2014–2020); in the MYR, negative (2000–2001), positive (2002–2016), negative (2017–2020); in the LYR, negative (2000–2005), positive (2006–2011), negative (2012–2014), positive (2015–2018), negative (2018–2020).

#### Two explanatory variables and one dependent variable

**Figure 5:**
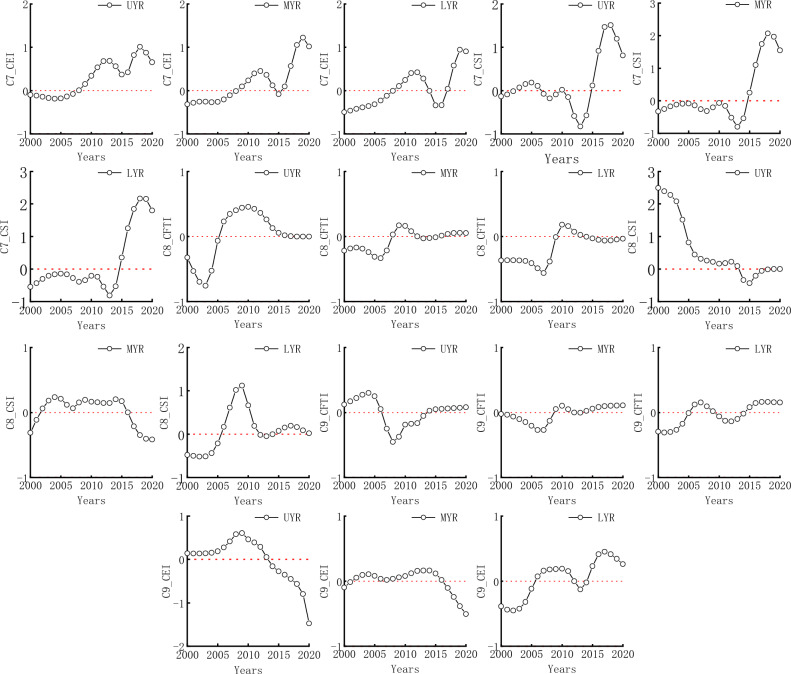
The impact of two explanatory variables on one dependent variable from 2000 to 2020.

For two explanatory variables and one dependent variable, Table 4 shows the other parameters. The feedback of different systems in the YRB from 2000 to 2020 is shown in Fig. 5. This model had good explanatory power and can effectively describe the relationship between two explanatory variables and one dependent variable. There is spatiotemporal heterogeneity between the three subsystems in the upper, middle, and lower reaches of the YRB and in terms of time.

**Table 4:** GTWR model results of capturing two of the three subsystems as explanatory variables and the other as dependent variables.

Dependent variable	CFTI	CEI	CSI
Explanatory variable	CEI and CSI (C7)	CFTI and CSI (C8)	CFTI and CEI (C9)
Bandwidth	0.115	0.115	0.115
Residual squares	0.699	0.225	0.077
Sigma	0.105	0.060	0.035
AICc	− 45.105	− 86.773	− 145.051
R^2^	0.685	0.758	0.967
Spatio-temporal distance ratio	4.889	1.557	1.357
Trace_of_SMatrix	18.707	24.572	26.129

The impact of CEI on CFTI shows a consistent trend in the UYR, MYR, and LYR, ranging from positive (2000–2007) to negative (2008–2018).

The impact of CSI on CFTI varies. In the UYR, there are negative (2000–2002), positive (2003–2006), negative (2007–2009), positive (2010), negative (2011–2014), and positive (2015–2020); in the MYR, negative (2000–2014), positive (2015–2020); in the LYR, negative (2000–2014), positive (2015–2020).

The impact of CFTI on CEI varied. In the UYR, there were negative (2000–2005), positive (2006–2017), and negative (2018–2020); in the MYR, negative (2000–2007), positive (2008–2012), negative (2013–2015), and positive (2016–2020); in the LYR, negative (2000–2009), positive (2010–2013), and negative (2014–2020).

The impact of CSI on CEI varied, with positive (2000–2013) and negative (2014–2020) in the UYR; in the MYR, negative (2000–2001), positive (2002–2016), negative (2017–2020); in the LYR, negative (2000–2005), positive (2006–2011), negative (2012–2014), and positive (2015–2020).

The impact of CFTI on CSI varied in the upper, middle, and lower reaches of the YRB, with negative (2000–2005), positive (2006–2011), negative (2013–2014), and positive (2015–2020) in the UYR; in the MYR, positive (2000–2006), negative (2007–2013), positive (2014–2020); in the LYR, negative (2000–2005), positive (2006–2009), negative (2010–2014), and positive (2015–2020).

The impact of CEI on CSI varied in the upper, middle, and lower reaches of the YRB, with positive (2000–2013) and negative (2014–2020) in the UYR; in the MYR, there were negative (2000–2001), positive (2002–2016), and negative (2017–2020); in the LYR, negative (2000–2005), positive (2006–2011), negative (2012–2014), and positive (2015–2020).

### The coupling coordination type of the Yellow River Basin

**Figure 6:**
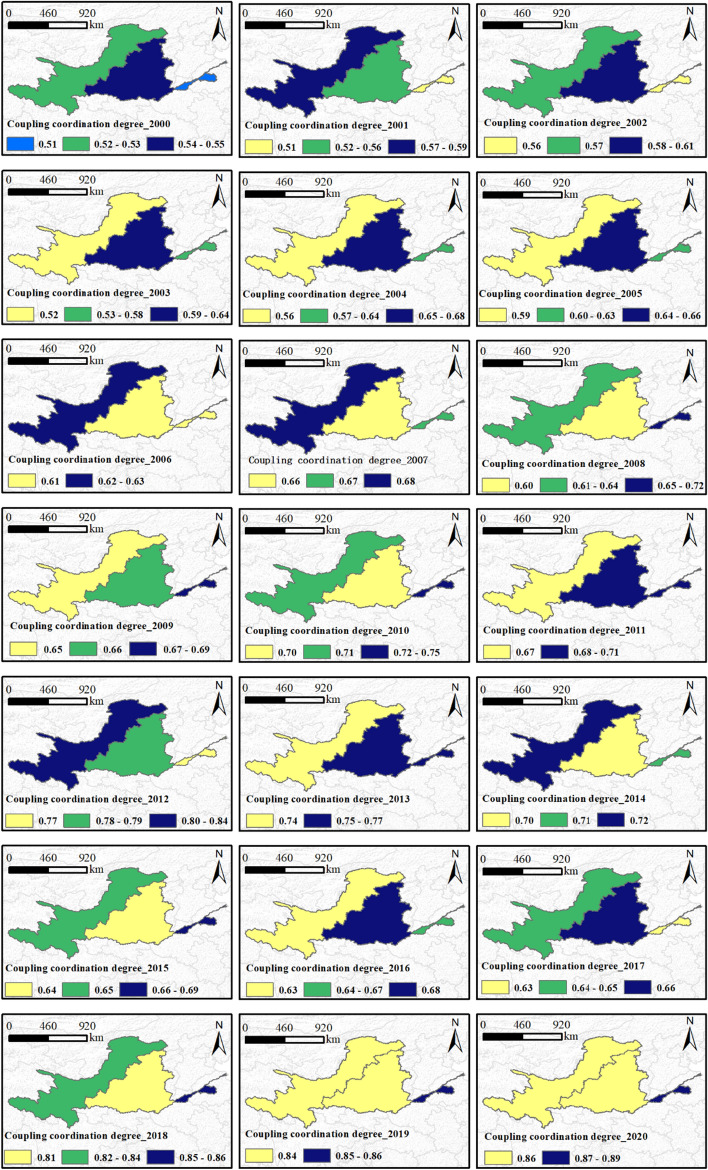
The CCD between FES along the YRB in China from 2000 to 2020. The map was generated by the authors with the help of ArcGIS 10.2 (https://support.esri.com/en/download/2093) and does not require any permission from anywhere.

Figure 6 shows the CCD variation trend of FES in the YRB's upper, middle, and lower reaches from 2000 to 2020. The map in Fig. 6 was prepared by the co-authors with the help of ArcGIS 10.2 (https://support.esri.com/en/download/2093). Overall, the CCD in the YRB indicated an increasing trend from reluctantly coordinated development to good coordinated development. In the UYR, CCD varied from 0.53 in 2000 to 0.862 in 2020, with levels ranging from reluctantly coordinated development (2000–2005), primary coordinated development (2006–2009, 2011, and 2015–2017), mode coordinated development (2010, 2013–2014), and good coordinated development (2018–2020). In the MYR, CCD varied from 0.56 in 2000 to 0.864 in 2020, with changes in levels ranging from reluctantly coordinated development (2000, 2003, and 2008), primary coordinated development (2001–2002, 2004–2006, 2010, 2013–2014, and 2016–2018), moderate coordinated development (2007, 2009, 2011 and 2015), and good coordinated development (2019–2020). In the LYR, CCD varied from 0.51 in 2000 to 0.89 in 2020, with levels ranging from reluctantly coordinated development (2000, 2002, 2004), primary coordinated development (2001, 2005, 2006, 2013, 2014, and 2016, 2018), moderate coordinated development (2007, 2011, 2015), and good coordinated development (2012, 2019, 2020).

**Figure 7:**
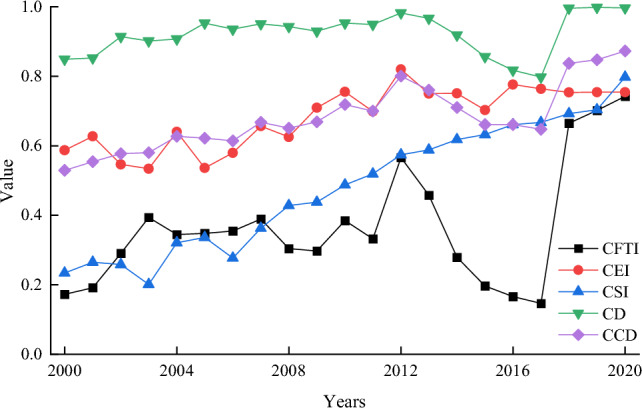
The change in average coordination degree between CFTI, CEI, and CSI in the Yellow River Basin of China from 2000 to 2020.

**Figure 8:**
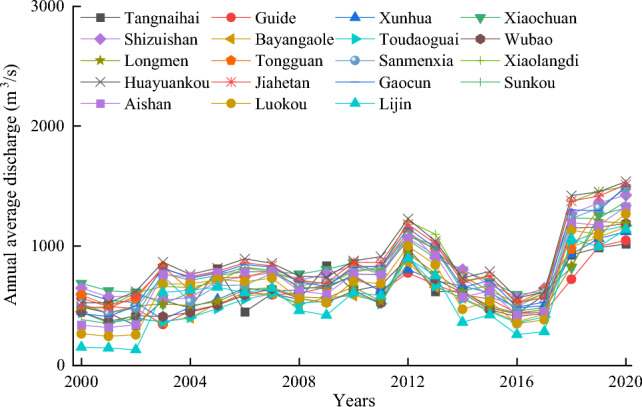
Characteristics of annual average discharge changes at major hydrological stations in the Yellow River Basin.

Figure 7 shows a significant improvement in CEI and CSI quality scores. CFTI fluctuates sharply between [0.17–0.74]. From 2012 to 2017, there was a downward trend in the values of various indicators in CFTI, leading to a decline in CFTI. Figure 8 shows the variation characteristics of the annual average discharge, which showed a significant downward trend from 2012 to 2017. The variation characteristics of other indicators are similar to Fig. 8. CFTI had a sharp decline from 2012 to 2017, mainly due to the decrease in variable internal indicators of CFTI.

CD demonstrated a downward trend from 2012 to 2017, but its average value remained above 0.92, slightly different from year to year. The CD value remains high, indicating a clear interaction between CFTI, CEI, and CUI. The range of CCD variation was 0.53–0.87, and the level can be divided into nine classes, namely reluctantly coordinated development with lagging flood media transportation (2000–2001), reluctantly coordinated development with lagging social-economic (2002–2003), primary coordinated development with lagging social-economic (2004–2007), immediate coordinated development with bagging flood edition transportation (2008–2009), modify coordinated development with bagging flood edition transportation (2010–2011), good coordinated growth with bagging flood edition transportation (2012), modify coordinated development with bagging flood edition transportation (2013–2014), primary coordinated development with lagging flood edition transportation (2015–2017), good coordinated development with lagging flood edition transportation (2018–2020). The lagging subsystems were flood and sediment transport (2000–2001 and 2008–2020) and socio-economy (2002–2007), with no lagging eco-environment.

### Analysis of the disparities in the CCD based on ESDA

**Figure 9:**
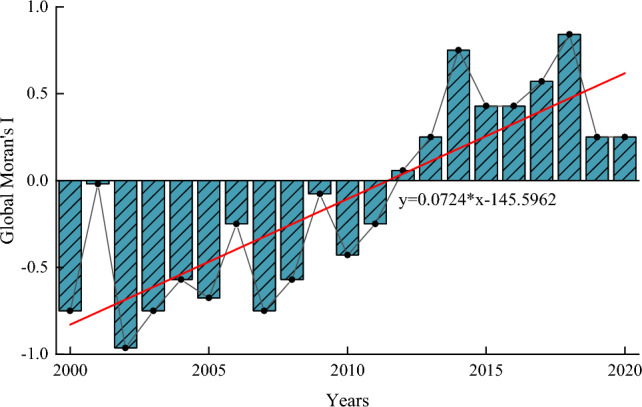
Moran scatter plot for the CCD along the YRB in China in 2000–2020.

Wang et al.^76^ pointed the CCD of an area depends not only on internal factors but also on the surrounding area. ESDA can confirm a trend of increasing or decreasing spatial bias. The spatial relationship characteristics of CCD in the study area were as follows (Fig. 9): The global Moran's I value of CCD from 2000 to 2011 was negative, showing negative spatial autocorrelation. However, from 2012 to 2020, CCD exhibited positive spatial autocorrelation. The global Moran's I changed from negative to positive, indicating a clustering trend in areas like CCD. The direction of spatial cohesion often increased over time.

## Discussion

### Evolutionary characteristics of various elements in the complex giant system of the Yellow River Basin

Systems are ubiquitous in nature and human society and can be classified into various types based on different principles^70^. Basin is a natural catchment area that is enclosed by the watershed of surface water and groundwater^77^. The basin is both a whole and has significant spatial heterogeneity, with complex and diverse forms of problems caused by the interaction between the river itself, the ecological environment, and socio-economic factors. At present, research on the elements of the Yellow River Basin system mainly includes water, energy and food^63^, low-carbon development and eco-environment^65^, sustainable development and environmental protection^66^, water utilization, industrial development and ecological welfare^67^, urbanization and water resource^78^, urbanization and ecosystem service value^79^, ecology and economy^80^, resources, economy and ecosystem^81^, human settlement environment and tourism industry^82^, public health, ecological environment and economic development^83^. agricultural carbon emissions efficiency and economic growth^84^, tourism, ecological-environment and public service^85^. This study found that the CCD values in the FES system of the Yellow River Basin showed an increasing trend, ranging from 0.53 to 0.87 for flood-sediment transportation, eco-environment, and socio-economy. Table 5 shows the CCD of other systems in the Yellow River Basin. In terms of time, relevant research mainly focuses on the past 20 years (2000–2020), with years as the time scale. The trend of CCD changes is mainly increasing, ranging from 0.26 to 0.87, with categories ranging from moderate functional decade to good coordinated development. These research results demonstrate that the relationships between various elements, such as ecology, economy, society, water resources, and population in the Yellow River Basin are becoming increasingly coordinated.

**Table 5:** CCD between different systems in the Yellow River Basin.

Basin	Year	System	CCD	References
Yellow River	2000–2020	Flood-sediment transportation, eco-environment and socio-economy	0.53–0.87	This study
	2000–2019	Water, energy and food	0.50–0.61	Wang et al.^63^
	2017–2019	Low-carbon development and eco-environment	0.66–0.82	Luo et al.^65^
	2000–2019	Sustainable development and environmental protection	0.26–0.85	Zhang et al.^66^
	2001–2020	Water utilization, industrial development and ecological welfare	0.31–0.33	Feng et al.^67^
	2008–2017	Urbanization and water resource	0.35–0.69	Qiao et al.^78^
	1995–2018	Urbanization and ecosystem service value	0.29–0.39	Zhang et al.^79^
	2011–2020	Ecology and economy	0.30–0.63	Zhao et al.^80^
	2000–2018	Resources, economy and ecosystem	0.57–0.81	Wang et al.^81^
	2010–2019	Human settlement environment and tourism industry	0.46–0.65	Yu and Chen^82^
	2009–2019	Public health, ecological environment and economic development	0.35–0.49	Wei et al.^83^
	2010–2020	Agricultural carbon emissions efficiency and economic growth	0.49–0.62	Qiao et al.^84^
	2008–2019	Tourism, ecological-environment and public service	0.50–0.53	Huang et al.^85^

### Construction of the index system

Based on the mutual feedback mechanism of the coupling system of flood and sediment transport, eco-environment, and socio-economy in the YRB, the goal was to promote the economic development of the beach area, ensure flood control safety, and maintain the ecological security of the basin. Based on previous research achievements and indicators, combined with the current research status, the principles of comprehensiveness, scientific, and typicality should be followed when selecting indicators^86^, mainly including (1) the principle of scientific, which can accurately express the relationship between social economy, flood control safety, and ecological environment; (2) the principle of comprehensiveness, starting from different perspectives, selects indicators that can represent the states of three systems, and the indicators of each subsystem are not included, repeated, or independent of each other; (3) the principle of hierarchy is to select indicators that are divided into first-level indicators and second level indicators; (4) the principle of dynamism is that all indicators change over time; (5) the principle of operability is that indicators have quantifiability and accessibility^87^. The socio-economic subsystem indicators drew on existing research^75^. The flood discharge and sand transport system indicators mainly adopted some basic characteristic indicators of the river, such as flow and sediment characteristics. The eco-environment indicators specifically considered the ecological water demand of key sections in the YRB.

### Measures for the coordinated development of FES

**Figure 10:**
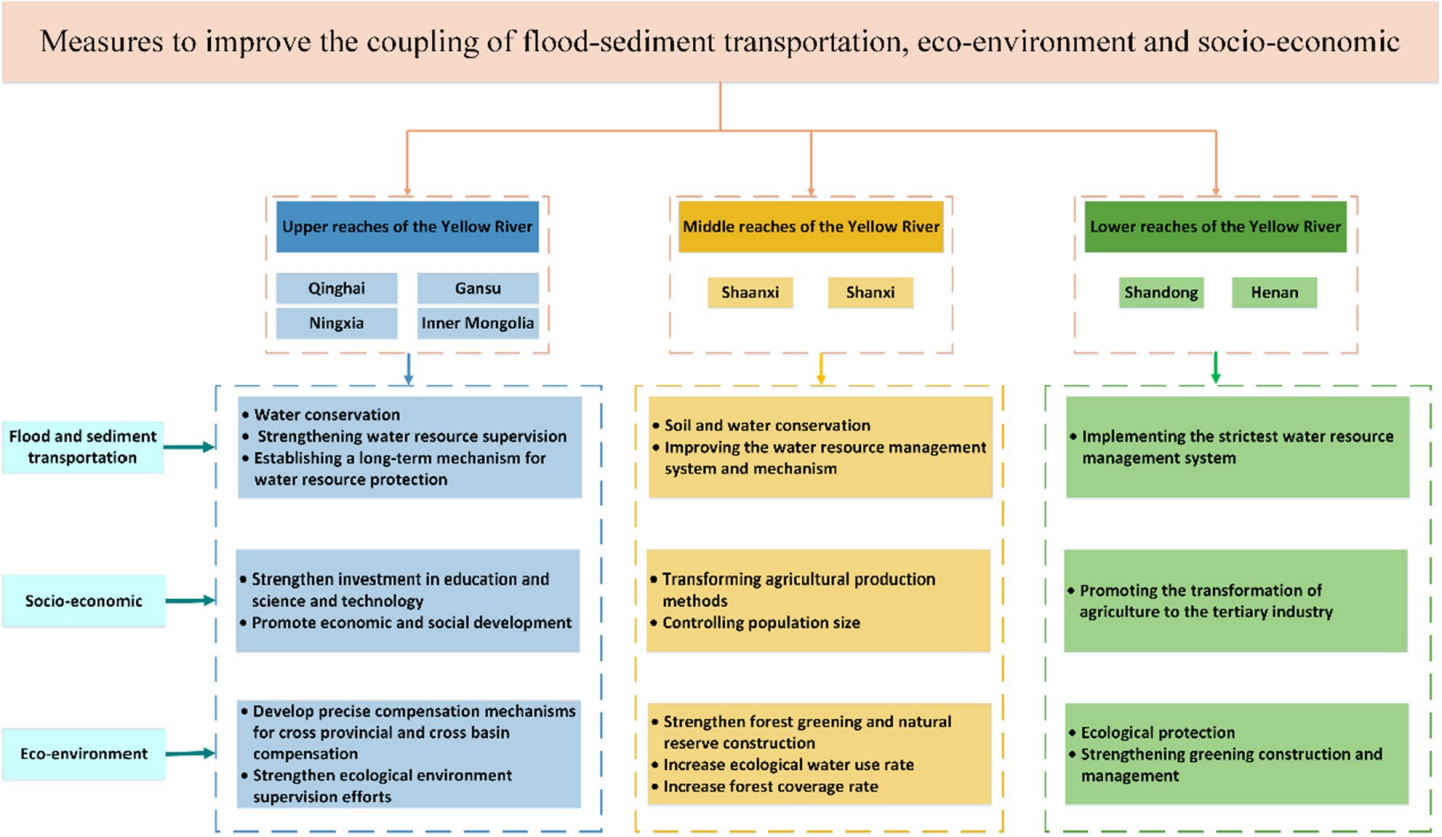
Regional collaboration and high-quality development path in the Yellow River Basin.

Based on the consumption and flow characteristics of water resources in the YRB, as well as the spatiotemporal evolution trend of coupled and coordinated development of flood-sediment transportation, eco-environment, and socio-economy, the study proposed an improvement path for regionally coordinated action in the YRB, which helps to improve the ecological protection and high-quality development level of the YRB (Fig. 10). UYR should strengthen its economic development, technological innovation, and resource optimization capabilities while protecting water conservation areas. MYR should further enhance new energy development and utilization capacity and strengthen ecological environment governance and restoration. LYR should adhere to concentrated and intensive development, enhance population and industrial carrying capacity, and fully leverage the leading role of high-quality economic development.

The measures that can be taken in flood-sediment transportation include water source conservation, strengthening water resource supervision, soil and water conservation, improving the water resource management system and mechanism, establishing a long-term mechanism for water resource protection, and implementing the strictest water resource management system. In terms of socio-economic aspects, measures can be taken to strengthen investment in education and science and technology, promote economic and social development, transform agricultural production methods, control population size, promote the transformation of agriculture to the tertiary industry, control population size, improve population quality, and adjust population structure. The measures that can be taken in terms of eco-environment include establishing precise compensation mechanisms for cross-provincial and cross-basin compensation, strengthening ecological environment supervision, strengthening forest greening and nature reserve construction, increasing ecological water use rate, increasing forest coverage, ecological protection, and strengthening green construction and management.

### Deficiencies and prospects

All models have certain uncertainties^88^. This study had some limitations and proposed possible future research directions. This study did not consider the failure to obtain some indicator data, mainly reflected in flood discharge, sediment transport, and ecological environment. The flood-sediment transportation subsystem included river hydrological processes, the carrying capacity of river channels, and the regulation capacity of reservoirs. The constraint factors can be divided into runoff and sediment, river boundary, and reservoir regulation capacity. This study only considered indicators related to runoff and sediment. The constraint factors in the eco-environment subsystem included river water quality, water surface area, habitat area, biodiversity, ecological water demand, vegetation coverage, etc. This study only considered the environmental flow needs of rivers, reflecting the survival environment, survival range, species structure, and the degree to which a series of requirements such as flow, water volume, and fluctuation timing are guaranteed and met during the critical development period of various organisms in the river system. Therefore, the incomplete indicator system, to some extent, affected the accuracy of the results. Thus, with future flood-sediment transportation and eco-environment data opening, it is necessary to enrich further and modify the established indicator system.

## Conclusion

The entropy methods, CCDM, GTWR, and ESDA, were used to analyze FES's spatial and temporal heterogeneity in China's YRB from 2000 to 2020. The main conclusions of this paper are as follows:

1. The CFTI and CEI in the YRB showed fluctuating growth, while CSI presented a linear growth trend. The CUI of various regions in the YRB constantly changed, with significant differences between regions. The CSI growth rate in Ningxia was as high as 36.03%, and overall, the quality of urbanization in the YRB was improving. The CFTI of Sanmenxia has the largest change, with a growth rate of 107.5%; The CEI of Toudaoguai has the largest change, with a growth rate of 6.48%.
2. Whether it was one explanatory variable and one dependent variable or two explanatory variables and one dependent variable, the positive and negative effects between FES alternate. There is heterogeneity in both time and space.
3. From 2000 to 2020, CCD in the upper, middle, and lower reaches of the YRB revealed an increasing trend. Still, the change rate differed from barely coordinated development to good coordinated development. The CCD variation range of the entire watershed ranged from 0.53 to 0.87, with the lagging subsystems being flood and sedimentation transport (2000–2001 and 2008–2020) and socio-economy (2002–2007), with no lagging eco-environment.
4. ESDA indicated that CCD in the YRB has shown different spatial clustering characteristics since 2000. From 2000 to 2011, CCD had negative spatial autocorrelation and tended to be distributed in space. From 2012 to 2020, CCD exhibited positive spatial autocorrelation and grew to be distributed spatially, reflecting the strengthening of connections between the UYR, MYR, and LYR. The above results reflect the spatial correlation and heterogeneity of CCD between FES in the YRB.

The research results provide powerful research tools and solid scientific support for the strategic layout and coordinated promotion of watershed system governance, protection, and high-quality socio-economic development.

## Data Availability

All data generated or analyzed during this study are included in this published article.

## Abbreviations

FES: Flood-sediment transportation, eco-environment, and socio-economy
YRB: Yellow River Basin
CFTI: Comprehensive flood-sediment transportation index
CEI: Comprehensive eco-environment index
CSI: Comprehensive socio-economy index
CCD: Coupling coordination degree
CD: Coupling degree
CCDM: Coupling coordination degree model
ESDA: Exploratory Spatial Data Analysis
GTWR: Geographically and Temporally Weighted Regression
UYR: Upper reaches of the Yellow River
MYR: Middle reaches of the Yellow River
LYR: Lower reaches of the Yellow River

## References

[1] United Nations World urbanization prospects: The 2022 revision (United Nations Department of Economic and Social Affairs (Population Division), 2022).

[2] Li KQ (2023). Government Work Report: Delivered at the Third Session of the 13th National People's Congress on May 13, 2023.

[3] China Rural Development Report 2020. (Institute of Rural Development, Chinese Academy of Social Sciences and China Social Sciences Press, 2020).

[4] Zhu YG, Ioannidis JPA, Li H, Jones KC, Martin FL (2011). Understanding and harnessing the health effects of rapid urbanization in China. Environ. Sci. Technol..

[5] Northam RM (1975). Urban Geography.

[6] Li YF, Li Y, Zhou Y, Shi YL, Zhu XD (2012). Investigation of a coupling model of coordination between urbanization and the environment. J. Environ. Manag..

[7] Tian L (2015). Land use dynamics driven by rural industrialization and land finance in the peri-urban areas of China: “The examples of Jiangyin and Shunde”. Land Use Policy.

[8] Fang CL, Wang ZB, Xu G (2016). Spatial-temporal characteristics of PM2.5 in China: A city-level perspective analysis. J. Geogr. Sci..

[9] Liu YS, Yan B, Zhou Y (2016). Urbanization, economic growth, and carbon dioxide emissions in China: A panel cointegration and causality analysis. J. Geogr. Sci..

[10] Wang SJ, Liu XP (2017). China's city-level energy-related CO_2_ emissions: Spatiotemporal patterns and driving forces. Appl. Energy.

[11] Li WF, Hai X, Han LJ, Mao JQ, Tian MM (2020). Does urbanization intensify regional water scarcity? Evidence and implications from a megaregion of China. J. Clean. Prod..

[12] Barbera E, Curro C, Valenti G (2010). A hyperbolic model for the effects of urbanization on air pollution. Appl. Math. Model..

[13] Wang QS, Yuan XL, Zhang J, Mu RM, Yang HC, Ma CY (2013). Key evaluation framework for the impacts of urbanization on air environment—A case study. Ecol. Ind..

[14] Wang ZB, Fang CL, Cheng SW, Wang J (2013). Evolution of coordination degree of eco-economic system and early-warning in the Yangtze River Delta. J. Geogr. Sci..

[15] Zhang JR, Zeng WH, Shi H (2016). Regional environmental efficiency in China: Analysis based on a regional slack-based measure with environmental undesirable outputs. Ecol. Ind..

[16] de Oliveira JAP, Doll CNH, Balaban O, Jiang P, Dreyfus M, Suwa A, Moreno-Penaranda R, Dirgahayani P (2013). Green economy and governance in cities: Assessing good governance in key urban economic processes. J. Clean. Prod..

[17] United Nations Development Programme (2016). UNDP support to the implementation of sustainable development goal 6.

[18] Cap-Net UNDP (2013). The 2nd Draft Strategy 2014–2017 “Water Knowledge for All”.

[19] Wei YL, Bao LJ, Wu CC, He ZC, Zeng EY (2015). Assessing the effects of urbanization on the environment with soil legacy and current-use insecticides: A case study in the Pearl River Delta, China. Sci. Total Environ..

[20] Liu YB, Li RD, Li CH (2005). Scenarios simulation of coupling system between urbanization and eco-environment in Jiangsu province based on system dynamic model. Chin. Geogr. Sci..

[21] Bao C, Fang CL (2015). Water resources constraint force on urbanization in water deficient regions: A case study of the Hexi Corridor, arid area of NW China. Ecol. Econ..

[22] Guan DJ, Gao WJ, Su WC, Li HF, Hokao K (2011). Modeling and dynamic assessment of urban economy-resource-environment system with a coupled system dynamics—geographic information system model. Ecol. Ind..

[23] Cardinale BJ, Duffy JE, Gonzalez A, Hooper DU, Perrings C, Venail P, Narwani A, Mace GM, Tilman D, Wardle DA, Kinzig AP, Daily GC, Loreau M, Grace JB, Larigauderie A, Srivastava DS, Naeem S (2012). Biodiversity loss and its impact on humanity. Nature.

[24] Liu YS, Fang F, Li YH (2014). Key issues of land use in China and implications for policy making. Land Use Policy.

[25] Chen Y, Syvitski JPM, Gao S, Overeem IL, Kettner AJ (2012). Socio-economic impacts on flooding: A 4000-year history of the Yellow River, China. Ambio.

[26] Li XN, Zhong DY, Zhang Y, Wang YJ, Wang YQ, Zhang HW (2018). Wide river or narrow river: Future river training strategy for Lower Yellow River under global change. Int. J. Sediment Res..

[27] Zhu JY, Song CQ, Wang JD, Ke LH (2020). China's inland water dynamics: The significance of water body types. Proc. Natl. Acad. Sci. USA.

[28] Zhang, H.Q. & Xu, E. Q. An evaluation of the ecological and environmental security on China's terrestrial ecosystems. *Sci. Rep.* **7**(l), 1–12 (2017). 10.1038/s41598-017-00899-x PMC5429794 28400605

[29] Xi JP (2019). Speech at the Symposium on ecological protection and quality development of the Yellow River basin China. Water Resour. Dev. Manag..

[30] Jin FJ (2019). Coordinated promotion strategy of ecological protection and high-quality development in the Yellow River Basin. Reform.

[31] He W, Lu YT (2022). A review and prospect on the studies of basin ecological economy. Ecol. Econ..

[32] Shen ZY, Ma YK, Feng CH, Liu RM, Chen L (2022). Review on evolution law and environmental effect of water and soil environment in watershed affected by human activities. Saf. Environ. Eng..

[33] Wang F, An LZ, Dang AR, Han JY, Miao CH, Wang J, Zhang GH, Zhao Y (2020). Past and future of land use change in the Middle reaches of the Yellow River Basin in China. Geogr. Res..

[34] Ives CD, Giusti M, Fischer J, Abson DJ, Klaniecki K, Dorninger C, Laudan J, Barthel S, Abernethy P, Martin-Lopez B, Raymond CM, Kendal D, von Wehrden H (2017). Human-nature connection: A multidisciplinary review. Curr. Opin. Environ. Sustain..

[35] Jiang EH, Wang YJ, Tian SM, Li JH, Xu LJ, Zhang XP (2020). Exploration of watershed system science. J. Hydraul. Eng..

[36] Guo YT, Wang HW, Nijkamp P, Xu JG (2015). Space-time indicators in interdependent urban-environmental systems: A study on the Huai River Basin in China. Habitat Int..

[37] Ariken M, Zhang F, Liu K, Fang CL, Kung HT (2020). Coupling coordination analysis of urbanization and eco-environment in Yanqi Basin based on multi-source remote sensing data. Ecol. Indic..

[38] Fang CL, Wang J (2013). A theoretical analysis of interactive coercing effects between urbanization and eco-environment. Chin. Geogr. Sci..

[39] Akie M, Fujisawa T, Sato T, Arai M, Saitoh K (2018). GeSn/SiGeSn multiple-quantum-well electroabsorption modulator with taper coupler for mid-infrared Ge-on-Si platform. IEEE J. Sel. Top. Quantum Electron..

[40] Li WW, Yi PT (2020). Assessment of city sustainability-coupling coordinated development among economy, society and environment. J. Clean. Prod..

[41] Liu JP, Tian Y, Huang K, Yi Y (2021). Spatial-temporal differentiation of the coupling coordinated development of regional energy-economy-ecology system: A case study of the Yangtze River Economic Belt. Ecol. Indic..

[42] Zhou D, Zhang XR, Wang XQ (2020). Research on coupling degree and coupling path between China's carbon emission efficiency and industrial structure upgrading. Environ. Sci. Pollut. Res..

[43] Ren Y (2011). On coordination development of agricultural ecological-environment and economy in Shaanxi Province based on coupling degree model. J. Arid Land Resour. Environ..

[44] Li LX, Cao XL, Wang P (2021). Optimal coordination strategy for multiple distributed energy system considering supply, demand, and price uncertainties. Energy.

[45] Tang Z (2015). An integrated approach to evaluating the coupling coordination between tourism and the environment. Tour. Manag..

[46] Mateusz T (2020). Analysing the coupling coordination degree of socio-economic-infrastructural development and its obstacles: the case study of Polish rural municipalities. Appl. Econ. Lett..

[47] Kong YD, Liu JG (2021). Sustainable port cities with coupling coordination and environmental efficiency. Ocean Coast. Manag..

[48] Fang CL, Liu HM, Li GD (2016). International progress and evaluation on interactive coupling effects between urbanization and the eco-environment. J. Geogr. Sci..

[49] Zhou HC, Li HJ, Chen XH, Zhu CF (2017). Analysis of coupling between high-speed railway and common speed railway system in transportation corridor. IOP Conf. Ser. Earth Environ. Sci..

[50] Dong SC, Zheng J, Li Y, Li ZH, Li FJ, Jin L, Yang Y, Bilgaev A (2019). Quantitative analysis of the coupling coordination degree between urbanization and eco-environment in Mongolia. Chin. Geogr. Sci..

[51] Lu CY, Yang JQ, Li HJ, Jin SL, Pang M, Lu CP (2019). Research on the spatial-temporal synthetic measurement of the coordinated development of population-economy-society-resource-environment (PESRE) systems in China based on geographic information systems (GIS). Sustainability.

[52] Chen JD, Li ZW, Dong YZ, Song ML, Shahbaz M, Xie QJ (2020). Coupling coordination between carbon emissions and the eco-environment in China. J. Clean. Prod..

[53] Wang JY, Wang SJ, Li SJ, Feng KS (2019). Coupling analysis of urbanization and energy-environment efficiency: Evidence from Guangdong province. Appl. Energy..

[54] Song M, Hu C (2018). A Coupling relationship between the eco-environment carrying capacity and new-type urbanization: A case study of the Wuhan metropolitan area in China. Sustainability.

[55] Xu WJ, Zhang XP, Xu Q, Gong HL, Li Q, Liu B, Zhang JW (2020). Study on the coupling coordination relationship between water-use efficiency and economic development. Sustainability..

[56] Dai HJ, Sun T, Zhang K, Guo W (2015). Research on rural nonpoint source pollution in the process of urban-rural integration in the economically-developed area in China based on the improved STIRPAT model. Sustainability.

[57] Yuan R, Zhao T, Xu XS, Kang JD (2015). Regional characteristics of impact factors for energy-related CO_2_ emissions in China, 1997–2010: Evidence from tests for threshold effects based on the STIRPAT model. Environ. Model. Assess..

[58] An L, Zvoleff A, Liu JG, Axinn W (2014). Agent-based modeling in coupled human and natural systems (CHANS): Lessons from a comparative analysis. Ann. Assoc. Am. Geogr..

[59] Pfeffer K, Verrest H, Poorthuis A (2015). Big data for better urban life?-An exploratory study of critical urban issues in two Caribbean cities: Paramaribo (Suriname) and Port of Spain (Trinidad and Tobago). Eur. J. Dev. Res..

[60] Shearmur R (2015). Dazzled by data: Big Data, the census and urban geography. Urban Geogr..

[61] Zheng Y (2015). Methodologies for cross-domain data fusion: An overview. IEEE Trans. Big Data.

[62] Kaginalkar A, Kumar S, Gargava P, Niyogi D (2021). Review of urban computing in air quality management as smart city service: An integrated IoT, AI, and cloud technology perspective. Urban Clim..

[63] Wang SS, Yang JY, Wang AL, Liu TF, Du SB, Liang ST (2023). Coordinated analysis and evaluation of water-energy-food coupling: A case study of the Yellow River basin in Shandong Province, China. Ecol. Indic..

[64] Wang YR, Song JX, Sun HT (2023). Coupling interactions and spatial equilibrium analysis of water-energy-food in the Yellow River Basin, China. Sustain. Cities Soc..

[65] Luo L, Wang YN, Liu YC, Zhang XW, Fang XL (2022). Where is the pathway to sustainable urban development? Coupling coordination evaluation and configuration analysis between low-carbon development and eco-environment: A case study of the Yellow River Basin, China. Ecol. Indic..

[66] Zhang KZ, Dong ZC, Guo L, Boyer EW, Liu JZ, Chen J, Fan BH (2023). Coupled coordination spatiotemporal analyses inform sustainable development and environmental protection for the Yellow River Basin of China. Ecol. Indic..

[67] Feng YJ, Zhu AK, Liu P, Liu ZL (2022). Coupling and coordinated relationship of water utilization, industrial development and ecological welfare in the Yellow River Basin, China. J. Clean. Prod..

[68] Li JS, Sun W, Li MY, Meng LL (2021). Coupling coordination degree of production, living and ecological spaces and its influencing factors in the Yellow River Basin. J. Clean. Prod..

[69] Swyngedouw E, Moulaert F, Rodriguez A (2002). Neoliberal urbanization in Europe: Large-scale urban development projects and the new urban policy. Antipode.

[70] Schumm SA (1977). The Fluvial System.

[71] Chu NC, Zhang PY, Wu XL (2022). Spatiotemporal evolution characteristics of urbanization and its coupling coordination degree in Russia-Perspectives from the population, economy, society, and eco-environment. Environ. Sci. Pollut. Res..

[72] Liao SJ, Wu Y, Wong SW, Shen LY (2020). Provincial perspective analysis on the coordination between urbanization growth and resource environment carrying capacity (RECC) in China. Sci. Total Environ..

[73] Wang XT (2021). Research on the Coupling Coordination of Economy-Recourse-Environment System in China.

[74] Huang B, Wu B, Barry T (2010). Geographically and temporally weighted regression for spatio-temporal modeling of house prices. Int. J. Geogr. Inf. Sci..

[75] Yang JY, Chen F, Wang YD, Mao JQ, Wang DL (2023). Performance evaluation of ecological transformation of mineral resource-based cities: From the perspective of stage division. Ecol. Indic..

[76] Wang Z, Wu W, Wu J (2005). An analysis to growth spillover cross regions in China. Geogr. Res..

[77] Zhao LJ, Li CM, Huang RB, Si S, Xue J, Huang W, Hu Y (2013). Harmonizing model with transfer tax on water pollution across regional boundaries in a China's lake basin. Eur. J. Oper. Res..

[78] Qiao R, Li HM, Han H (2021). Spatio-temporal coupling coordination analysis between urbanization and water resource carrying capacity of the provinces in the Yellow River Basin, China. Water.

[79] Zhang KL, Liu T, Feng RR, Zhang ZC, Liu K (2021). Coupling coordination relationship and driving mechanism between urbanization and ecosystem service value in large regions: A case study of urban agglomeration in Yellow River Basin China. Int. J. Environ. Res. Public Health.

[80] Zhao YH, Hou P, Jiang JB, Zhai J, Chen Y, Wang YC, Bai JJ, Zhang B, Xu HT (2021). Coordination study on ecological and economic coupling of the Yellow River Basin. Int. J. Environ. Res. Public Health.

[81] Wang SS, Yang JY, Wang AL, Yan YF, Liu TF (2022). Coupled coordination of water resources-economy-ecosystem complex in the Henan section of the Yellow River basin. Water Supply.

[82] Yu X, Chen HX (2022). Study on coupling coordination of the human settlement environment and tourism industry in the Yellow River basin. Front. Environ. Sci..

[83] Wei W, Jin CG, Han Y, Huang ZH, Niu T, Li JK (2022). The coordinated development and regulation research on public health, ecological environment and economic development: Evidence from the Yellow River Basin of China. Int. J. Environ. Res. Public Health.

[84] Qing Y, Zhao BJ, Wen CH (2023). The coupling and coordination of agricultural carbon emissions efficiency and economic growth in the Yellow River Basin, China. Sustainability.

[85] Huang ZH, Wei W, Han Y, Ding SY, Tang K (2022). The coupling coordination evolutionary analysis of tourism-ecological environment-public service for the Yellow River Basin of China. Int. J. Environ. Res. Public Health.

[86] Liu YB, Yao CS, Wang GX, Bao SM (2011). An integrated sustainable development approach to modeling the eco-environmental effects from urbanization. Ecol. Indic..

[87] Guo Q (2021). Research on Water Ecological Carrying Capacity of Taiyangshan Wetland Based on Weber-Fechner Model.

[88] Teklay A, Dile YT, Asfaw DH, Bayabil HK, Sisay K, Ayalew A (2022). Modeling the impact of climate change on hydrological responses in the Lake Tana basin, Ethiopia. Dyn. Atmos. Oceans.

